# P-156. Global burden of mortality and antimicrobial resistance of pathogens associated with peritoneal and intraabdominal infection in 2019: A Benchmarking Global Analysis

**DOI:** 10.1093/ofid/ofae631.361

**Published:** 2025-01-29

**Authors:** Ishita Hirapara, Nkechi Enemuo, Lovekumar Vala, Kavya Maddineni, Parthkumar Nandania, Keethanshan Markandu, Dhruvi Modi, Hardik Dineshbhai Desai

**Affiliations:** G.M.E.R.S. Medical College, Junagadh, Gujarat, India; Fairfield General Hospital., Bury, England, United Kingdom; Department of Internal Medicine, Shantabaa Medical College and General Hospital, Amreli, Gujarat, India, 365601, Amreli, Gujarat, India; College of Public Health, Kent State University, Kent, Ohio, USA, 44242, Kent, Ohio; Jonelta Foundation School of Medicine, University of Perpetual Help System DALTA, Las Piñas- 1750, NCR, Philippines, Las pinas, National Capital Region, Philippines; Public Health Sciences, Penn State College of Medicine (Pennsylvania State University), Hershey, Pennsylvania, USA, PA 17033, Hershey, Pennsylvania; Gujarat Adani Institute of Medical Sciences, Bhuj, Gujarat, India; Gujarat Adani Institute of Medical Sciences, Affiliated K.S.K.V University, Ahmedabad, Gujarat, India

## Abstract

**Background:**

Peritoneal and abdominal infections (PAI) are among the most serious complications in the clinical setting, often leading to significant morbidity and mortality worldwide. These infections, which can stem from a variety of pathogens, are further complicated by the increasing threat of antimicrobial resistance (AMR).

Deaths per 100k both associated with and attributable to bacterial antimicrobial resistance by pathogen Peritoneal & abdominal infection, Global, Age-standardized, Both sexes, 2019

**Figure:**
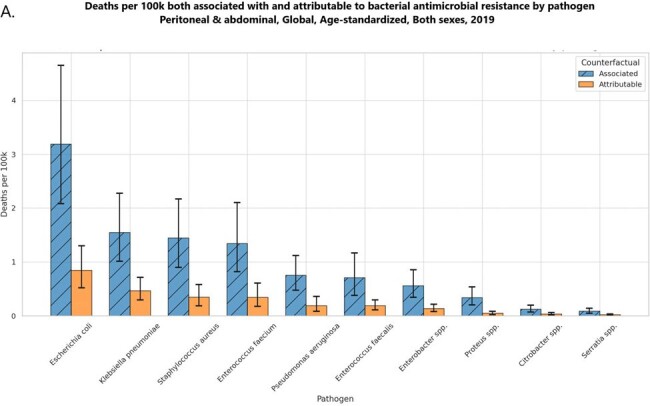

**Methods:**

Using Global Burden of Disease (GBD) AMR 2019 methodology, we estimated the number of deaths and the disability-adjusted life years (DALYs) globally attributable to and associated with AMR of pathogens associated with PAI in 2019.

DALYs per 100k both associated with and attributable to bacterial antimicrobial resistance by pathogen Peritoneal & abdominal infection, Global, Age-standardized, Both sexes, 2019

**Figure:**
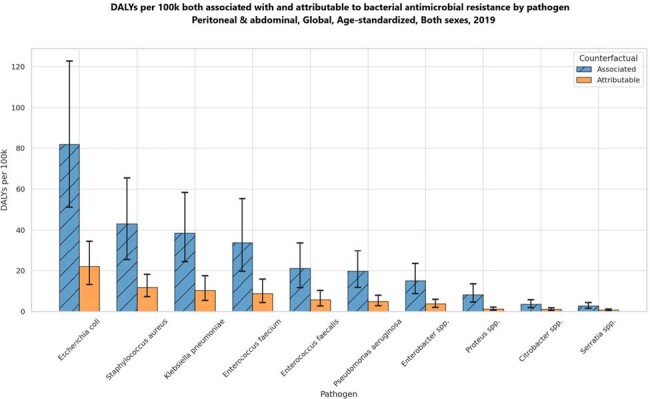

**Results:**

The data reveals that in 2019, E. coli was the leading cause of AMR-associated deaths in the context of PAI, with an estimated 289,206 deaths (95% uncertainty interval [UI]: 187,670-423,363), followed by S. aureus with 169,222 deaths (95% UI: 105,231-252,817). Among the countries studied, Romania reported the highest mortality rate from E. coli associated with PAI at 8.45 per 100,000 population, followed closely by Bulgaria at 7.99, while the United States recorded a lower rate of 5.17. Additionally, the highest number of deaths linked to AMR overall was observed with E. coli (252,952 deaths), followed by K. pneumoniae (123,158), S. aureus (115,616), and E. faecium (107,226) in 2019.

Total DALYs per 100k by pathogen Peritoneal & abdominal infection, Global, Age-standardized, Both sexes, 2019

**Figure:**
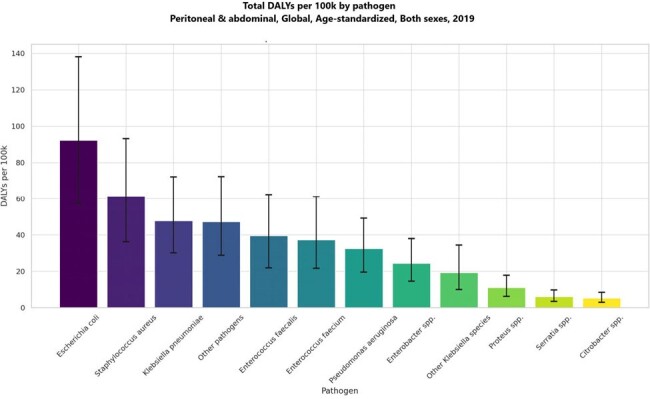

**Conclusion:**

The findings of this study underscore the urgent need for enhanced public health measures to combat the escalating threat of antimicrobial resistance (AMR) associated with peritoneal and abdominal infections. Public health stakeholders must prioritize the development and implementation of robust surveillance systems, promote rational use of antibiotics, and invest in research to discover new antimicrobial agents. From a clinician's perspective, there is a critical need for adherence to stringent infection control practices, timely and accurate diagnosis, and tailored treatment strategies to effectively manage these infections and mitigate the burden of AMR. Together, these actions are essential for improving patient outcomes and curbing the global rise in antibiotic-resistant pathogens.

Total deaths per 100k by pathogen Peritoneal & abdominal infection, Global, Age-standardized, Both sexes, 2019

**Figure:**
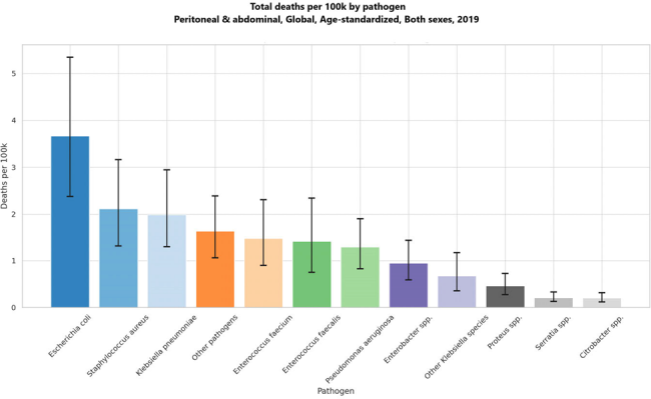

**Disclosures:**

**All Authors**: No reported disclosures

